# Book Review

**DOI:** 10.1120/jacmp.v4i2.2535

**Published:** 2003-03-01

**Authors:** Walter Huda

**Affiliations:** ^1^ Department of Radiology SUNY Upstate Medical University 750 E. Adams St. Syracuse NY 13210

## Abstract

**Applied Imaging Technology.** 4th ed. J. C. P. Heggie, N. A. Liddell, and K. P. Maher. St. Vincent's Hospital, Melbourne, Australia, 2001. http://www.life.rmit.edu.au/mrs/kpm/AIT/

PACS number(s): 87.57.

*Applied Imaging Technology* is a textbook written for use by radiology residents in Australasia. The book is approximately 500 pages long, and is divided into the following major sections: Radiation Biology & Safety; Basic Physics of X‐ray Imaging; Technology of X‐ray Imaging; Magnetic Resonance; Nuclear Medicine; and Ultrasound. This publication is concise, comprehensive, and current. In addition, the technical quality of the text and illustrations is high. *Applied Imaging Technology* reflects the needs of Australasia in terms of radiation regulations and radiologic practice. Nonetheless, I think this is an excellent book to supplement the standard texts on medical imaging found in many European and North American radiology departments. This book includes a wealth of up‐to‐date references to the scientific literature as well as more than 500 multiple choice questions. Anyone involved in teaching radiology residents and technologists should be able to benefit from the numerous drawings and illustrations in this book, as well as the medical images which enhance the reader's understanding of the accompanying text.

